# Computational modeling of human vagus nerve stimulation with three-dimensional fascicular morphology

**DOI:** 10.1063/5.0308450

**Published:** 2026-03-02

**Authors:** Daniel P. Marshall, Aniruddha R. Upadhye, Ozge N. Buyukcelik, Andrew J. Shoffstall, Warren M. Grill, Nicole A. Pelot

**Affiliations:** 1 Department of Biomedical Engineering, Duke University, Durham, North Carolina 27708, USA; 2 Department of Biomedical Engineering, Case Western Reserve University, Cleveland, Ohio 44106, USA; 3 Department of Electrical and Computer Engineering, Duke University, Durham, North Carolina 27708, USA; 4 Department of Neurosurgery, Duke University School of Medicine, Durham, North Carolina 27708, USA; 5 Department of Neurobiology, Duke University School of Medicine, Durham, North Carolina 27708, USA

## Abstract

Implanted vagus nerve stimulation is FDA-approved to treat epilepsy, depression, and stroke sequelae and is under development for other disorders such as heart failure and rheumatoid arthritis. Anatomically realistic computational models enable the design of electrodes and stimulation parameters that activate nerve fibers that mediate therapeutic responses, and avoid activating fibers that cause side effects. Conventional modeling techniques assume constant longitudinal morphology, extruding a single cross section to define the three-dimensional nerve geometry. However, recent imaging data showed that human vagus nerves have extensive fascicle splitting and merging along their length. Therefore, we developed a pipeline to simulate true three-dimensional (true-3D) models of peripheral nerve stimulation from segmentations of micro-computed tomography imaging. We implemented models of n = 4 human vagus nerves and systematically evaluated extrusion vs true-3D model responses to electrical stimulation across population dose-response relationships, fiber-specific thresholds, recruitment order, and spatial selectivity. Despite the complex morphology of the human vagus nerve, extrusion models replicated the true-3D neural responses if: (1) the nerve morphology was deformed to a circular cross section, as occurs with chronic cuff implants, and (2) the extruded cross section was centered under the depolarizing electrode contact. Our pipeline provides a foundation for advanced modeling of peripheral nerve stimulation and the design of more selective stimulation therapies.

## INTRODUCTION

Implanted cervical vagus nerve stimulation (VNS) is FDA-approved to treat epilepsy,^1^ depression,^2^ and stroke sequelae.^3^ VNS is under investigation for other indications, including treatment of heart failure^4^ and rheumatoid arthritis.^5,6^ The dominant side effects of VNS—voice alteration, coughing, and hoarseness—are caused by activation of off-target nerve fibers that form the recurrent laryngeal nerve that branches off of the vagus nerve.^7–10^ Clinical stimulation amplitudes are generally selected to avoid intolerable side effects from the activation of these off-target fibers; however, this often leads to inadequate activation of target fibers that mediate therapeutic responses.^7,8,11,12^ Selective stimulation that activates target fibers while avoiding activation of off-target fibers is needed.^13^ Computational modeling provides a powerful approach to design selective VNS as an important complement to *in vivo* and clinical studies; compared to these methods, models can evaluate a much broader range of design permutations.

Model-based design of VNS can increase the ratio of activated on-target to off-target nerve fibers by leveraging the topographical organization of the cervical vagus nerve, i.e., the spatial segregation of these fiber groups.^14–18^ Typically, the morphology of a modeled nerve is defined by extruding a two-dimensional segmented histological or synthetic cross section.^19–23^ However, the morphology of the human cervical vagus nerve changes rapidly along its length, with fascicles splitting or merging every 0.56 mm on average.^24^ Therefore, the standard extrusion approach—which does not account for any longitudinal variations in morphology—may not be appropriate for modeling human VNS. Several morphological features that impact activation thresholds are not captured by an extrusion model, including fiber curvature,^25^ fascicle interconnections resulting in a single endoneurium volume, and longitudinal variations in nerve diameter, electrode-fiber distance, and fascicle diameter.^26,27^

We developed a novel approach to model electrical stimulation of peripheral nerves using true three-dimensional (true-3D) nerve morphology. These models were built using segmentations of micro-computed tomography (microCT) imaging of human vagus nerves; the models thus capture the nerves' anatomical complexity, including merges and splits of fascicles. Our pipeline presents several innovations in finite element modeling of peripheral nerve stimulation: (1) curvilinear coordinates to calculate anisotropic conductivities of the 3D endoneurium, an important advance since true-3D models require a non-constant vector for the direction of anisotropy; (2) structural mechanics to model deformation of the 3D nerve morphology in response to chronic cuff instrumentation, a new technique since prior models excluded deformation or only applied deformation to 2D nerve cross sections; and (3) 3D fiber paths through the fascicular structure, a necessity given the complex morphology of true-3D models.

We used our novel modeling pipeline to compare the responses of true-3D vs extrusion models of human VNS, including population responses, individual fiber thresholds, recruitment order, spatial patterns of activation, and selective activation of target fibers across several domains. We quantified responses for different stimulation cuff electrodes, nerve deformation conditions, and selections of nerve cross sections for extrusion. Specifically, we evaluated two different cuff electrodes for delivering stimulation to compare nonselective, circumneural stimulation delivered via the standard clinical cuff vs localized stimulation delivered via a multi-contact cuff. We modeled true-3D and extrusion models with and without deformation of the nerve that occurs with chronic cuff electrode implantation. Finally, we quantified the sensitivity to selection of the nerve cross section used for building the extrusion model: the center of each clinical cuff contact and the center between the two contacts. Unexpectedly, under certain conditions the patterns of neural activation in extrusion models agreed with those from true-3D models regardless of stimulation cuff: specifically, when we deformed the nerve morphology to the cuff electrode and when we used the nerve cross section at the cathode-leading stimulation pulse for extrusion.

## RESULTS

**FIG. 1.**
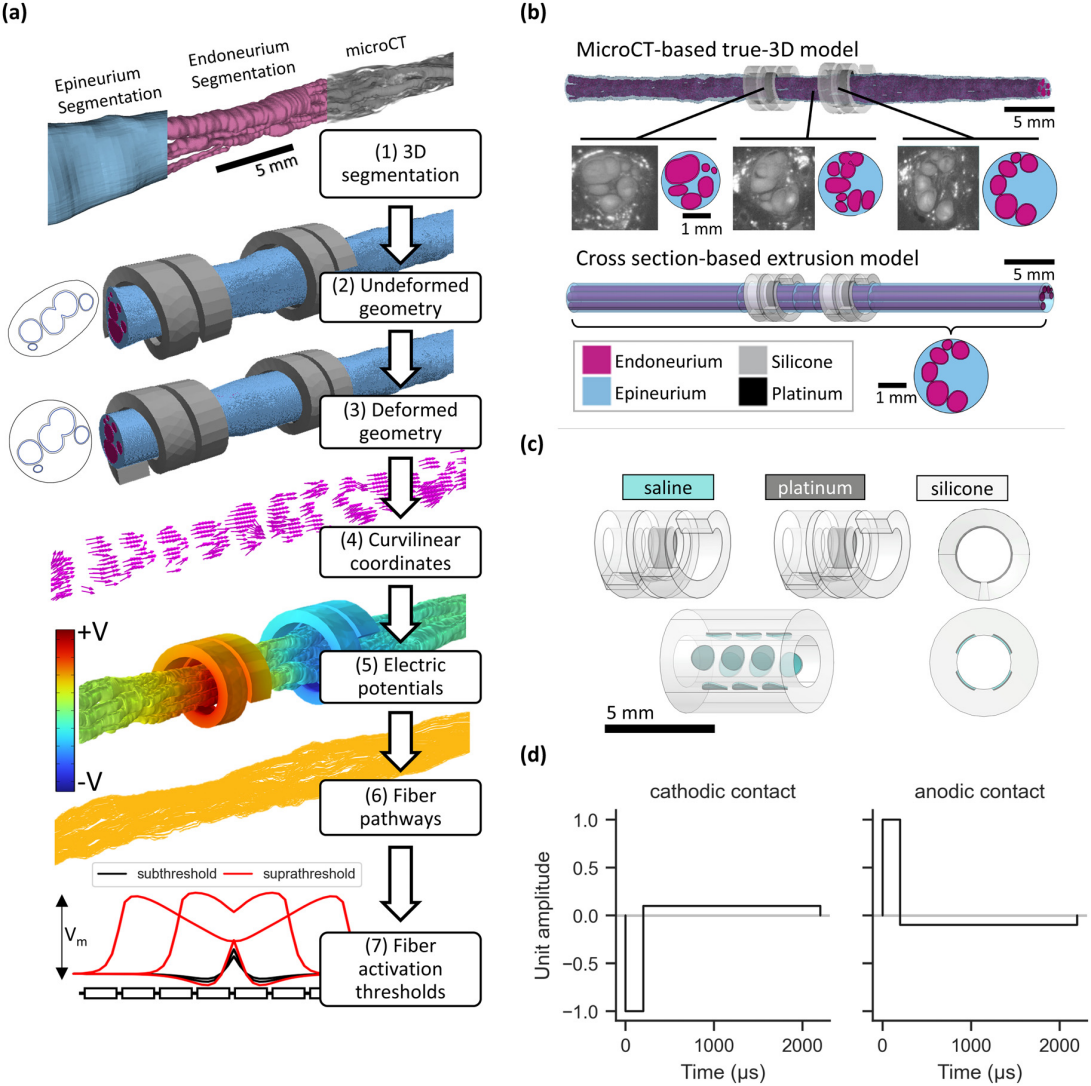
Methods for modeling peripheral nerve stimulation using true-3D and extrusion models. (a) Flow chart for modeling peripheral nerve stimulation with true-3D morphology, using example images for sample 6R. From top to bottom: (1) The original microCT volumetric image, alongside segmentation of the endoneurium and epineurium. The scale bar applies to all subpanels except (7). Model of the segmented 3D nerve and clinical VNS cuff, before (2) and after (3) deformation, alongside the nerve cross section under the cathodic contact (not to scale). (4) Arrows showing the vectors for curvilinear coordinates throughout the endoneurial domain. Curvilinear coordinates are used to define the anisotropic conductivity of the endoneurium when solving for the electric potentials and to define the fiber pathways. (5) Electrical potentials from bipolar stimulation calculated throughout the model; only the endoneurium is shown. (6) Fiber pathways throughout the endoneurium calculated from the curvilinear coordinates. (7) Example of action potential generation and propagation for a suprathreshold stimulus (red) and depolarization without action potential generation for a subthreshold stimulus (black). (b) Example true-3D model (top) and extrusion model (bottom), shown for sample 6R. The morphology of the true-3D model changes along its length, as seen in the example raw and segmented microCT images. The morphology of the extrusion model is constant for all slices; in this example, we used a cross section from the true-3D morphology located under the center of one of the electrode contacts. The models also included perineurium, saline around each cuff, and surrounding muscle (not shown). A detailed description of the model geometry and parameters is given in Methods: Finite element models of electric currents. (c) Cuff geometries used for computational models. The bipolar circumneural cuff electrode (top) is the standard device used clinically to treat epilepsy and depression, shown with a resting inner diameter of 2 mm. The 12-contact cuff electrode (bottom) is a custom geometry created for this study, shown with a resting inner diameter of 2 mm. We also modeled both cuffs with resting inner diameters of 3 mm (not shown) as needed for larger nerves; the clinical cuff expanded to accommodate nerves larger than its resting inner diameter by unfurling, while the multi-contact cuff opened with a longitudinal slit in its insulation. Instrumentation of the cuff on the nerve is described in “Cuff geometry,” and the assignment of contacts for electrical stimulation is described in Fig. 3(a) for the circumneural cuff and Fig. 8(b) for the multi-contact cuff. (d) Charge-balanced biphasic stimulation waveform, with a 200 *μ*s primary phase, followed by a 2 ms recharge phase. For the circumneural cuff, the cathodic contact was the caudal helix, and the anodic contact was the rostral helix. Note that this differs from clinical VNS, in which the cathodic-leading pulse is delivered via the rostral contact.

We used our novel computational pipeline [Fig. 1(a)] to simulate activation thresholds for human VNS using microCT-based true-3D nerve morphologies [Fig. 1(b)]. For each true-3D model, we used the ASCENT open-source pipeline^22^ to implement three extrusion models using (1) the “cathodic” slice from the center of the cathode-leading contact, (2) the “anodic” slice from the center of the anode-leading contact, and (3) the “center” slice from between the two contacts. For true-3D models, we either left the nerve undeformed or used structural mechanics to shape it to conform to the cuff electrode; for extrusion models, we likewise created undeformed models using slices from the undeformed true-3D models, as well as deformed models either using slices from the deformed true-3D models (3D deformation) or through 2D deformation applied to slices from the undeformed true-3D models (2D deformation). In total, we implemented 8 true-3D nerve morphologies (4 nerve samples × 2 deformation conditions) and 36 extruded nerve morphologies (4 nerve samples × 3 slices × 3 deformation conditions). We modeled each nerve morphology with a bipolar circumneural cuff and a custom 12-contact electrode placed at the same location as the cathode-leading circumneural contact. Unless otherwise stated, we used the circumneural cuff [Fig. 1(c)] with a charge-balanced biphasic asymmetric waveform [Fig. 1(d)]. For most visualizations, we focused on 3 and 13 *μ*m diameter fibers, as these represent the primary modes of the fiber diameter distribution in the vagus nerve^28–30^ and represent putative therapeutic and side effect-mediating fibers, respectively.

### The fascicular morphology of the human vagus nerve is complex and varies along its length

**FIG. 2.**
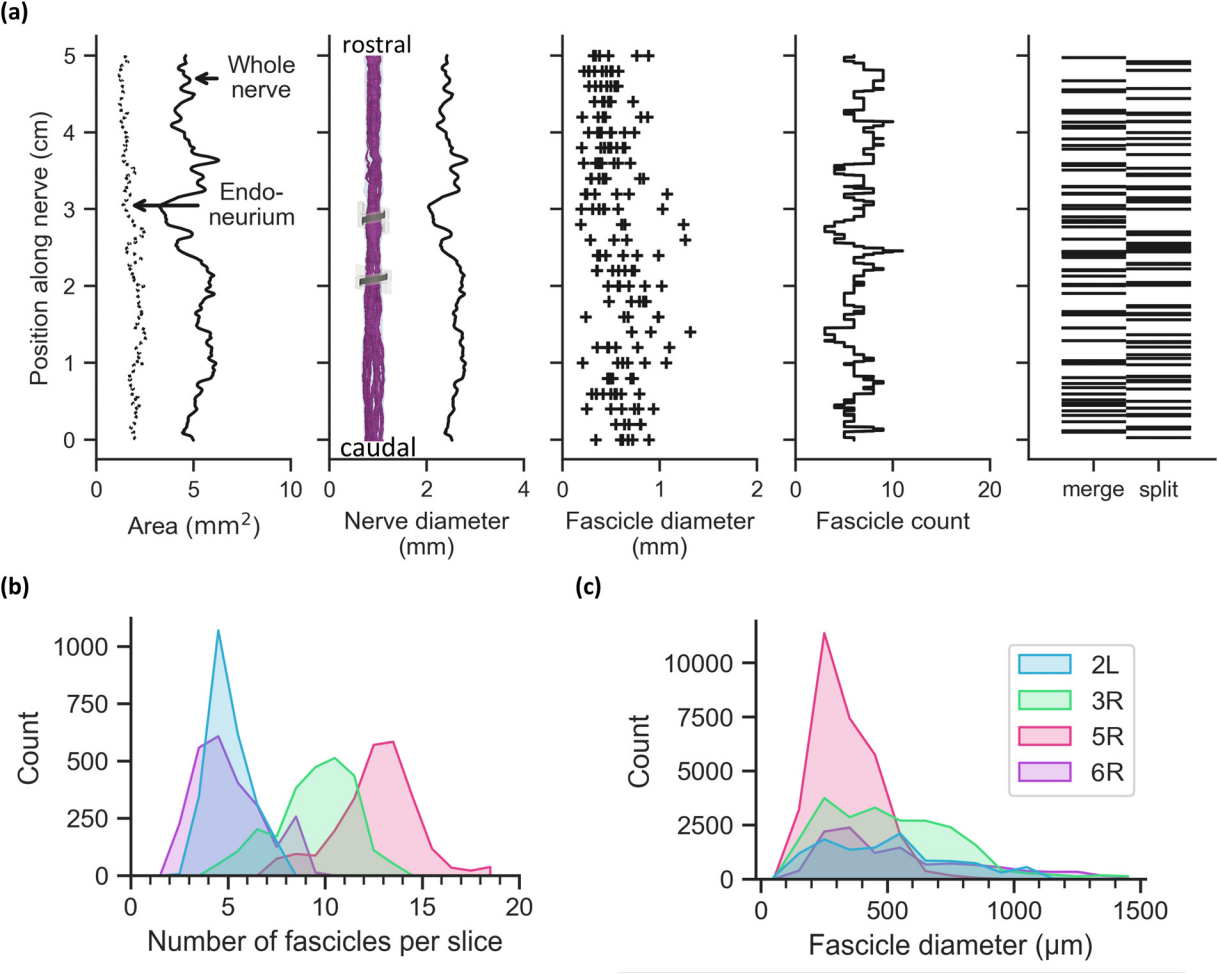
Morphology of the human cervical vagus nerve. Diameter (of nerve or fascicle) refers to effective circular diameter [Eq. (2)]. L and R refer to nerves from the left and right sides, respectively. (a) Longitudinal morphology for sample 6R; plots for all nerves are provided in Supplement 7. Fascicle diameters are plotted every 2 mm along the nerve. (b, c) Distributions of per-slice fascicle counts (b) and effective circular diameters (c) for each nerve, calculated from slices every 20 *μ*m (2504 slices after de-stepping each 5.01 cm-long nerve sample). Ticks on the x axis denote the histogram bins.

The number and size of fascicles in the human cervical vagus nerve changed rapidly along the nerve length [Fig. 2(a); Supplement 7]. The four nerves had varied distributions of fascicle counts per slice [Fig. 2(b); median of 7 fascicles per slice across all slices in all nerves; range of 2–19] but similar distributions of fascicle diameters [Fig. 2(c); median effective circular diameter of 384 *μ*m across all fascicles in all slices in all nerves; range of 44–1557 *μ*m]. The fascicles split or merged on average (mean) every 725, 350, 249, and 448 *μ*m for samples 2L, 3R, 5R, and 6R, respectively.

### Appropriately parameterized extrusion models replicate dose-response properties of true-3D models

We compared dose-response curves of extrusion and true-3D models by calculating the proportion of active fibers as a function of stimulation amplitude. Extrusion model thresholds to activate 10%, 50%, or 90% of fibers (i.e., onset, half, and saturation, respectively) corresponded well to true-3D model thresholds when (1) the nerve was deformed to conform to the cuff electrode and (2) the extrusion model used the nerve slice under the cathodic contact.

**FIG. 3.**
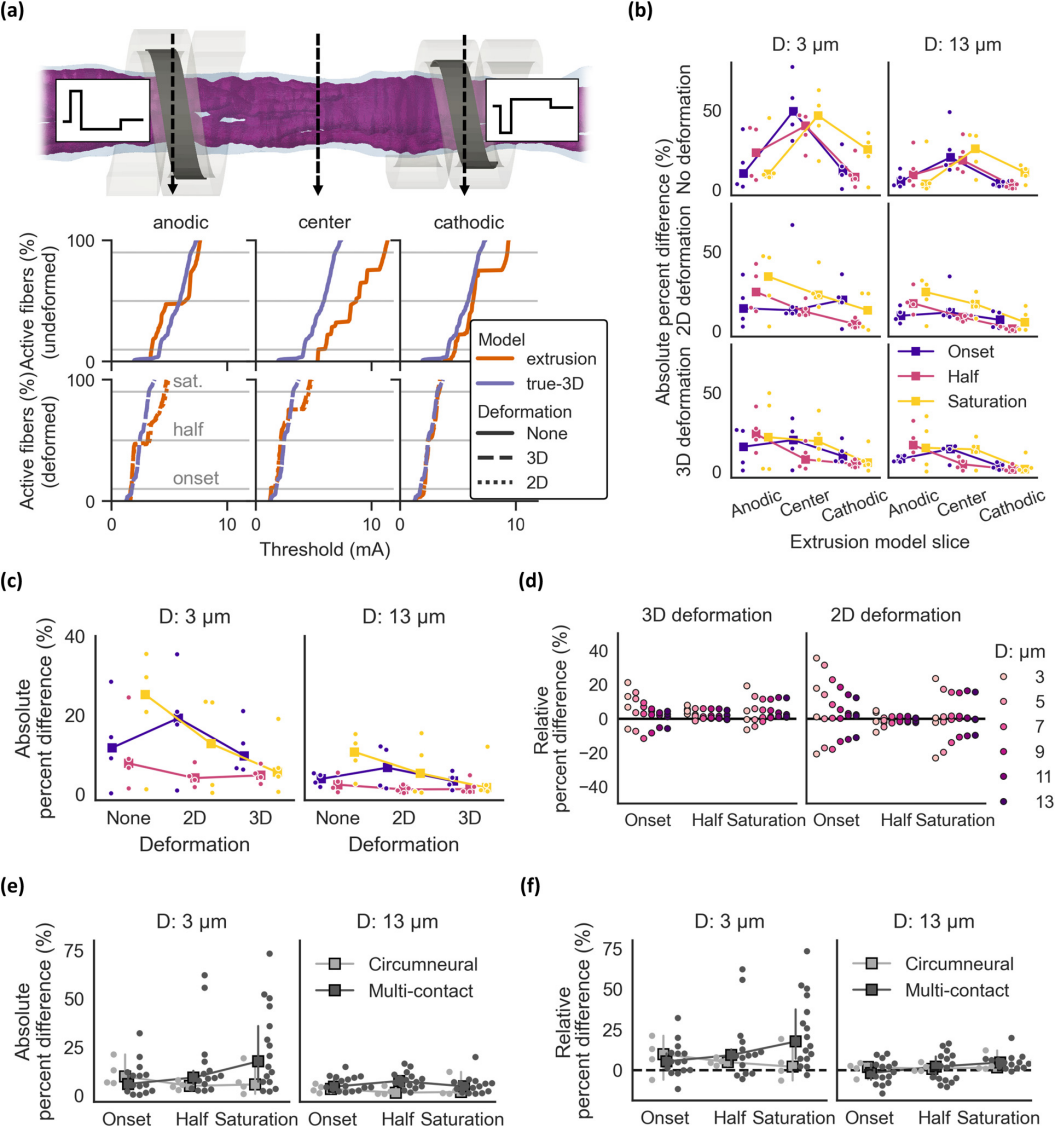
Dose-response thresholds for human VNS with n = 4 samples modeled as true-3D and extruded morphologies. Relative and absolute percent differences were calculated between extrusion and true-3D model thresholds to activate 10% (onset), 50% (half), and 90% (saturation) of fibers. The relative percent difference is the difference between the extrusion and true-3D thresholds, divided by the true-3D threshold; therefore, values >0 indicate higher thresholds for extrusion models. The absolute percent difference is the absolute value of the relative percent difference. In plot titles and legends, “D: <value> *μ*m” indicates the fiber diameter. (a) Dose-response curves for 3 *μ*m fibers in sample 3R. Data for all samples are in Supplement 8. Curves are shown for models with no deformation applied, extrusion and true-3D models with 3D deformation, and extrusion models with 2D deformation. (b, c) Absolute percent difference across extrusion slices and deformation conditions for 3 and 13 *μ*m fibers (b) and across deformation conditions for cathodic-slice extrusion models (c) (square markers = median). For the “2D deformation” case, data are compared between 2D-deformed extrusion models and 3D deformed true-3D models. (d) Relative percent difference across threshold levels and fiber diameters for deformed samples and cathodic-slice extrusion models. (e, f) Absolute (e) and relative (f) percent difference between onset, half, and saturation thresholds for 3D deformation models using the circumneural or multi-contact cuff (square markers = median; error bars = 95% confidence interval obtained via bootstrapping). For each sample using the multi-contact cuff, there are 4 data points (total of 16 points across all 4 samples), one for each contact used for stimulation (cathodic monopole with each contact in the center row).

Without deformation, threshold differences between the true-3D and extrusion models were inconsistent across the nerve slices used to define the extrusion models [Fig. 3(a), top row; Fig. 3(b), top row, Supplement 8]. Deformation decreased thresholds [Fig. 3(a), bottom row vs top row; Supplement 8] due to a reduction in electrode-fiber distances and space between the cuff and nerve; this space is filled with saline that shunts current away from the nerve. We deformed true-3D models using structural mechanics and compared them to extrusion models using slices from the deformed true-3D models (3D deformation) or 2D physics (2D deformation). For both 2D and 3D deformed extrusion models, extrusion slices further from the cathodic contact generally resulted in greater threshold differences from deformed true-3D models [Fig. 3(b), middle and bottom rows]; therefore, the analyses in the remainder of this section use the cathodic slice for extrusion models.

In cathodic-slice extrusion models, the agreement between true-3D and extrusion model thresholds was better with deformation (2D or 3D), with one exception: the difference in onset currents was smaller for the undeformed models than for the 2D-deformed extrusion models [Fig. 3(c)]. Overall, the percent difference in activation thresholds between true-3D and extrusion models was lowest when using 3D deformation (median absolute percent difference for onset, half, and saturation of 9.7%, 4.8%, and 5.5%, respectively, for small-diameter fibers and 3.3%, 1.3%, and 1.6% for large-diameter fibers). Across all comparisons, the match was better for larger-diameter fibers [Figs. 3(c) and 3(d)] and for half thresholds [Fig. 3(d)]. On average, extrusion models had higher thresholds than paired true-3D models [Fig. 3(d)].

We compared thresholds between extrusion and true-3D models using circumneural vs multi-contact cuffs. For the multi-contact cuff, we calculated thresholds with each of the four contacts on the center row as an active cathodic monopole. For both cuffs, large-diameter fiber thresholds matched well between extrusion and true-3D models [Fig. 3(e); median absolute percent difference of 4.7% and maximum of 19.7% across cuffs, activation levels, active contacts, and nerves]. Small-diameter fiber half and saturation thresholds had larger percent differences with the multi-contact cuff for some nerves and active contacts [Fig. 3(e); median of 10.5% and maximum of 73.1%]. As with the circumneural cuff, multi-contact cuff extrusion models had higher thresholds than paired true-3D models, particularly for smaller fibers [Fig. 3(f)].

Overall, extrusion models using the slice under the cathodic contact had dose-response thresholds that matched well the true-3D dose-response thresholds after deformation from chronic instrumentation with a cuff electrode.

### Cathodic-slice extrusion models have the best correspondence of individual fiber thresholds to true-3D models

**FIG. 4.**
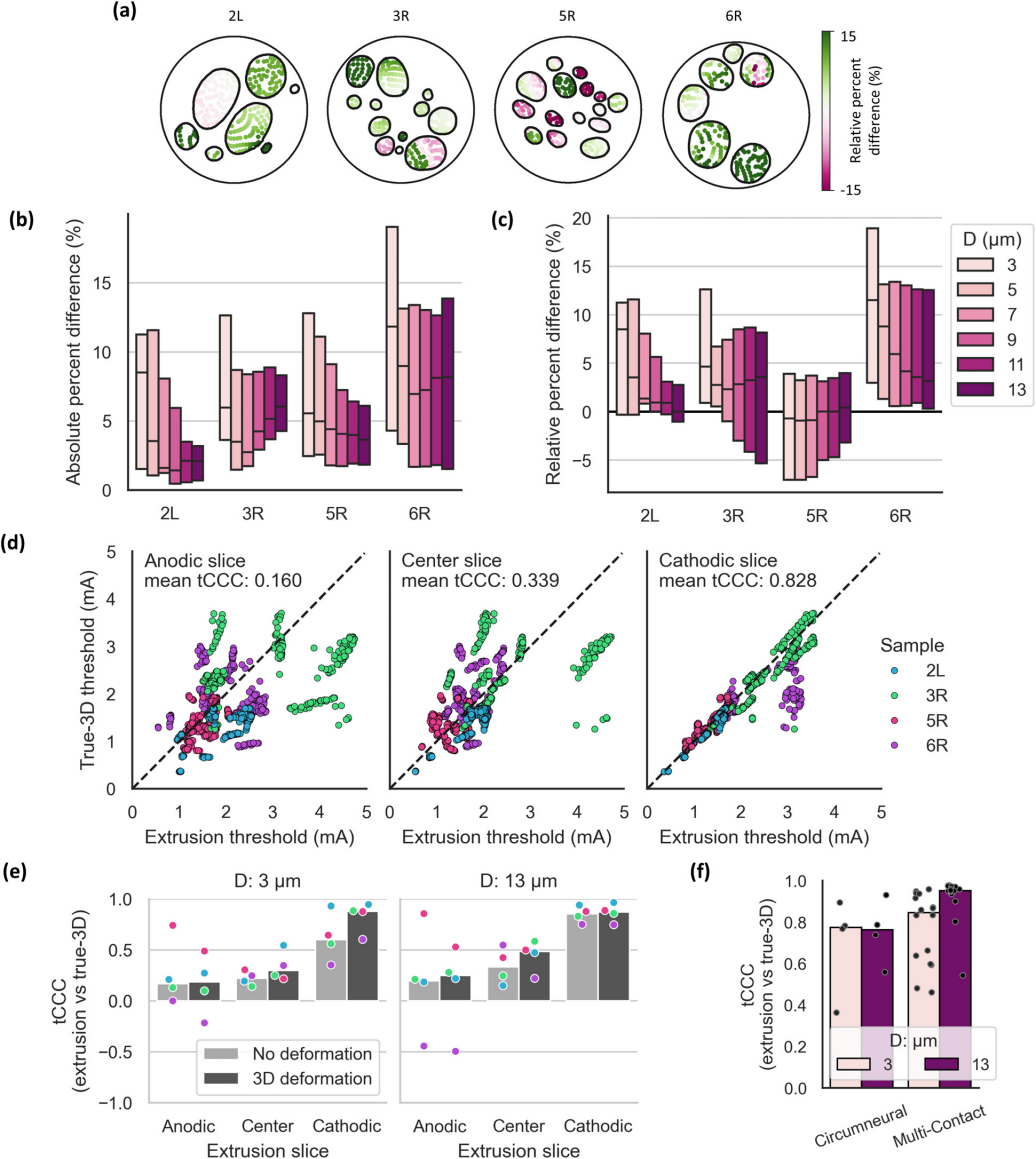
Comparisons of modeled thresholds across fiber locations matched between the true-3D and extrusion models. We used the cathodic slice and 3D deformation for extrusion models unless otherwise specified. (a) Relative percent difference between true-3D and extrusion model threshold for each fiber in each sample. The colorbar extends from −15% to 15%; values outside this range are assigned the closest limit. (b, c) Absolute (b) and relative (c) percent differences between thresholds from extrusion and true-3D models calculated for each fiber in each nerve. Boxplots show median and interquartile range. All data points are shown in Supplement 9. (d) True-3D model thresholds vs extrusion model thresholds for 3 *μ*m fibers, where each fiber location is one point, overlaid with a y = x line. We calculated the threshold concordance correlation coefficient (tCCC) for each sample; each subpanel title has the mean across the four samples. (e) tCCC between true-3D and extrusion thresholds for each sample [dots with colors as in panel (d), bars = median]. (f) tCCC between true-3D and extrusion thresholds for each sample with circumneural and multi-contact cuffs (bars = median). For each sample using the multi-contact cuff, there are 4 data points (total of 16 points), one for each contact used for stimulation (cathodic monopole with each contact in the center row).

We next compared thresholds for individual fiber locations between true-3D and extrusion models. Differences in fiber thresholds varied throughout each nerve; the differences were often homogeneous within a fascicle or distinctly clustered within a fascicle, reflecting nearby fascicle branching [Fig. 4(a)]. Across all fiber diameters and nerves, median absolute percent differences of fiber thresholds in 3D deformed, cathodic-slice extrusion models vs true-3D models were <12% [Fig. 4(b)]; 90% of absolute percent differences in threshold were <20% for 3 *μ*m fibers (maximum of 150%) and <12% for 13 *μ*m fibers (maximum of 26%). As with the dose-response thresholds [Figs. 3(d) and 3(f)], the difference generally decreased as fiber diameter increased [Fig. 4(b)], and thresholds were higher in extrusion models for most nerves and fiber diameters [Fig. 4(c)].

We used Lin's concordance correlation coefficient^31^ to compare the fiber-specific thresholds (tCCC) between extrusion and true-3D models. Consistent with the dose-response curves [Fig. 3(b)], the match between individual fiber thresholds in true-3D and extrusion models was best when using the cathodic slice [Fig. 4(d)]. For the cathodic-slice extrusion models, deformation increased tCCC for small-diameter fibers but did not affect the concordance for large-diameter fibers [Fig. 4(e)]. For large-diameter fibers, tCCC was a better match for models using the multi-contact cuff than those using the circumneural cuff [Fig. 4(f)].

### Cathodic-slice extrusion models have the best correspondence of activation order across fiber locations to true-3D models

**FIG. 5.**
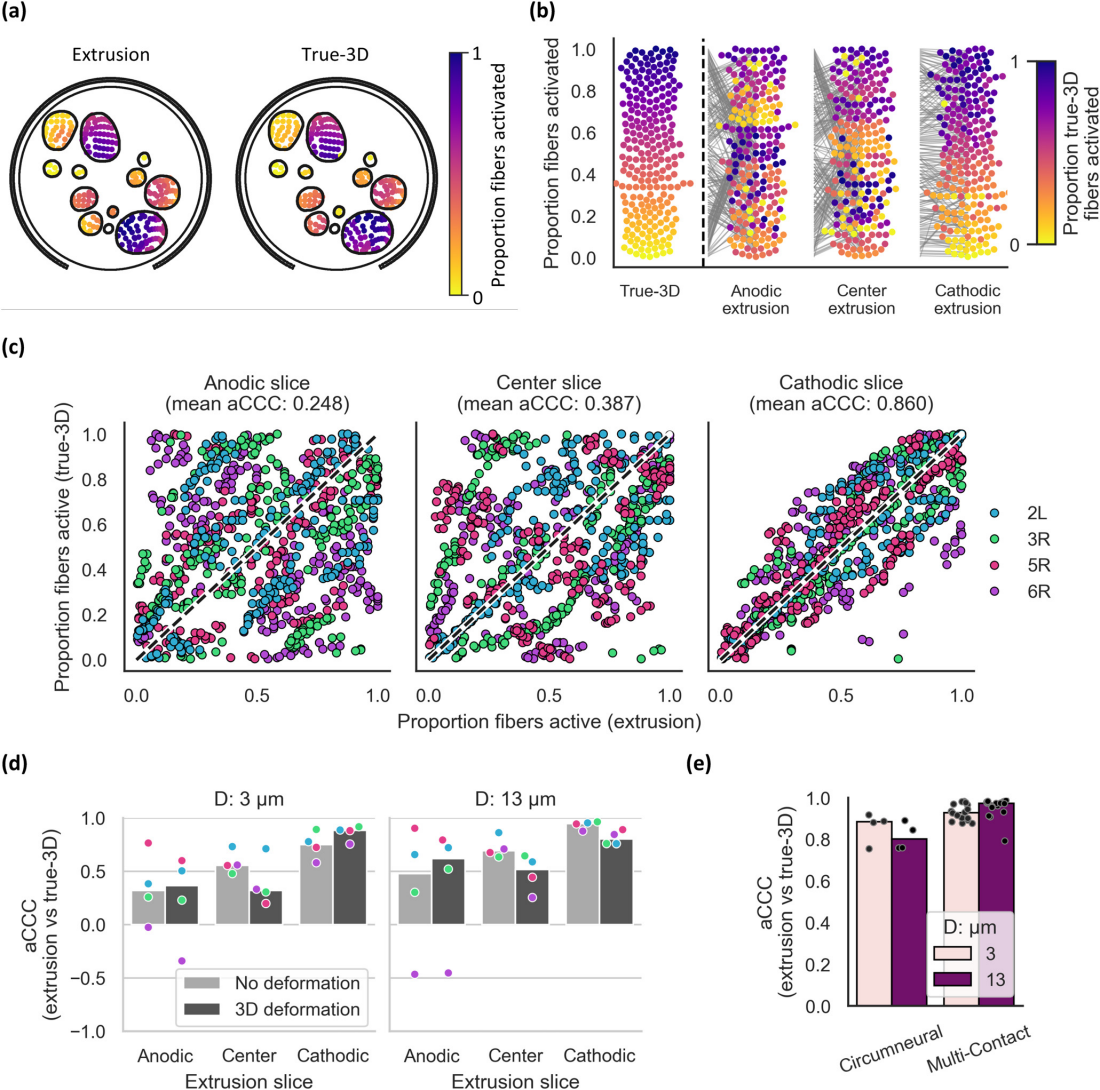
Differences in recruitment order across fiber locations matched between the deformed true-3D and extrusion models. (a) Heatmap of activation order for each fiber for 3 *μ*m fibers in sample 3R. Extrusion thresholds are from the cathodic 3D deformation model. Plots for all nerves are provided in Supplement 10. To assign fiber colors for true-3D and extrusion models, we calculated the proportion of fibers activated at or below each fiber's threshold. (b) Recruitment order for 3 *μ*m fibers in sample 3R, color-coded by the true-3D model recruitment order. Each fiber is represented as a point, where the stimulation amplitude required to activate that fiber will also activate all fibers below it. Gray lines show the change in values between true-3D and extrusion models. Plots for all nerves are provided in Supplement 11. (c) True-3D model recruitment order vs extrusion model recruitment order for 3 *μ*m fibers, where each fiber location is one point, overlaid with a y = x line (dashed line). We calculated the activation concordance correlation coefficient (aCCC) for each sample; each subpanel title has the mean across the four samples. (d) aCCC values between true-3D and extrusion thresholds for each sample [dots with nerve-specific colors as in panel (c), bars = median]. (e) aCCC values between true-3D and cathodic-slice extrusion models across 4 nerve samples simulated with one (circumneural) or four (multi-contact) contact configurations (dots) per sample (bars = median). For each sample using the multi-contact cuff, there are 4 data points (total of 16 points), one for each contact used for stimulation (cathodic monopole with each contact in the center row).

Differences in fiber-specific thresholds between extrusion and true-3D models (Fig. 4) may change the order in which fibers are activated as the stimulation amplitude is increased (i.e., recruitment order). Therefore, for each model, we calculated the activation order across fiber locations [Figs. 5(a) and 5(b)], and we quantified the fiber recruitment order in true-3D vs extrusion models by calculating the concordance correlation coefficient on the activation order values (aCCC).

Figure 5(b) shows activation order across fiber locations for an example true-3D model compared to corresponding extrusion models. In this example and when we calculated aCCC between true-3D models for all nerves and extrusion slices [Fig. 5(c)], fiber recruitment order in true-3D vs extrusion models was most similar for the cathodic-slice extrusion models. Unlike other metrics, there was little influence of deformation on fiber recruitment order [Fig. 5(d)]. The aCCC values were higher for the multi-contact cuff compared to the circumneural cuff [Fig. 5(e)]. Overall, extrusion models reproduced well the fiber recruitment order from true-3D models if the cathodic slice was used for extrusion.

### Activation order for different fiber diameters differs more in true-3D models than in extrusion models

**FIG. 6.**
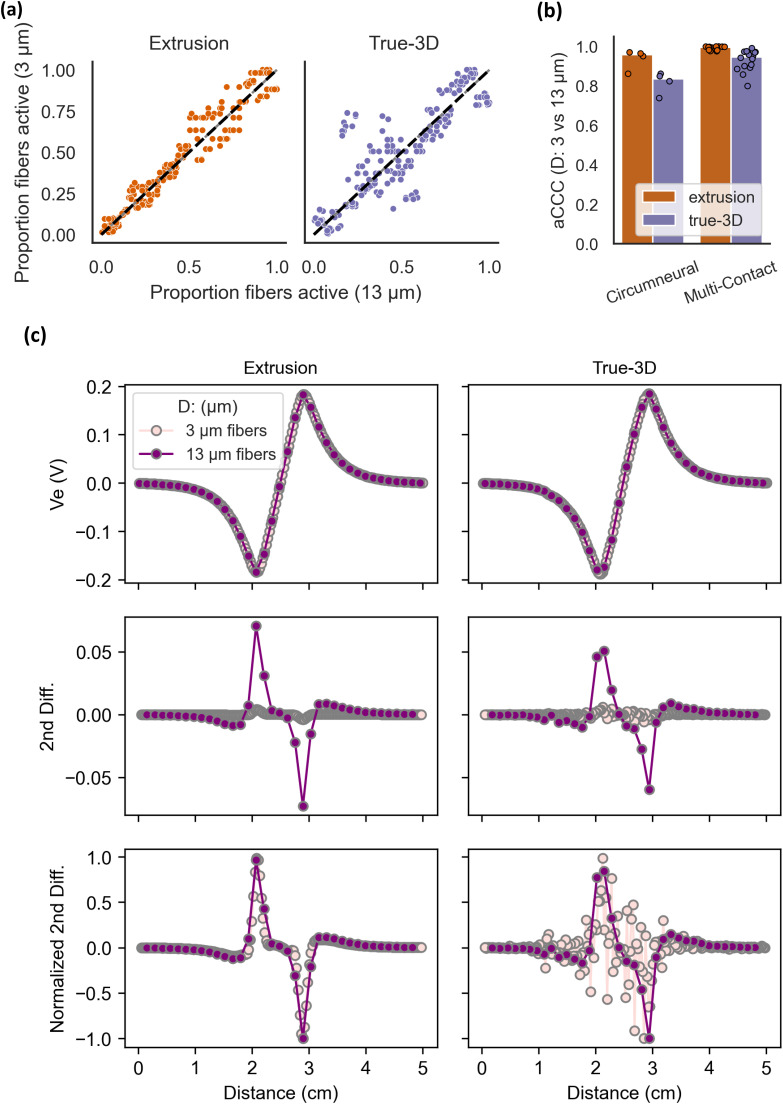
Differences in fiber location recruitment order for small vs large fiber diameters in extrusion (orange) and true-3D (purple) models. We randomly jittered the longitudinal alignment of each fiber by 0 to half its internodal length. (a) Recruitment order of 3 vs 13 *μ*m fibers across fiber locations for deformed extrusion (cathodic slice) and true-3D models of sample 5R. Data for all samples are given in Supplement 12. (b) aCCC values between 3 and 13 *μ*m fibers for cathodic 3D deformation models using both cuffs (bars = median). For each sample using the multi-contact cuff, there are 4 data points (total of 16 points), one for each contact used for stimulation (cathodic monopoles on each contact in the center row). (c) Extracellular potentials (V_e_) and their second difference, for example 3 and 13 *μ*m diameter fibers (on the same fiber path) from sample 5R. Extracellular potentials were sampled at the nodes of Ranvier for bipolar stimulation with a 1 mA stimulus. Plots for all fibers in all nerves are given in Supplement 16.

We quantified differences in activation order between small and large fiber diameters across fiber locations within a given true-3D or extrusion model [Fig. 6(a)]. Without true-3D morphology, the expectation from neural biophysics would dictate that fiber locations are generally recruited in the same order for any given fiber diameter. Indeed, extrusion models had aCCC values near 1 [median aCCC = 0.957 for the circumneural cuff; Fig. 6(b)], predicting that fiber recruitment order is highly similar across diameters. Conversely, in true-3D models, varying fiber diameter changed the order in which fibers were activated [median aCCC = 0.836 for the circumneural cuff; Fig. 6(b)]. The aCCC was even higher using the multi-contact electrode, and it was again higher for extrusion models [0.996 for extrusion vs 0.946 for true-3D; Fig. 6(b)].

Changes in transmembrane potential are driven by the second spatial difference of the extracellular potentials sampled at the nodes of Ranvier,^32^ which are more closely spaced for smaller fibers. While extrusion model fibers had smooth second difference curves across fiber diameters, local potential variations in true-3D models cause jagged second difference curves for small-diameter fibers; these potential variations are downsampled by the larger spacing between large-diameter fiber nodes of Ranvier, which is a likely cause of differential activation patterns across fiber diameters [Fig. 6(c)]. In summary, we found that the recruitment order of fiber locations differed more across fiber diameters for true-3D models.

### Thresholds vary more within fascicles for true-3D models than extrusion models

**FIG. 7.**
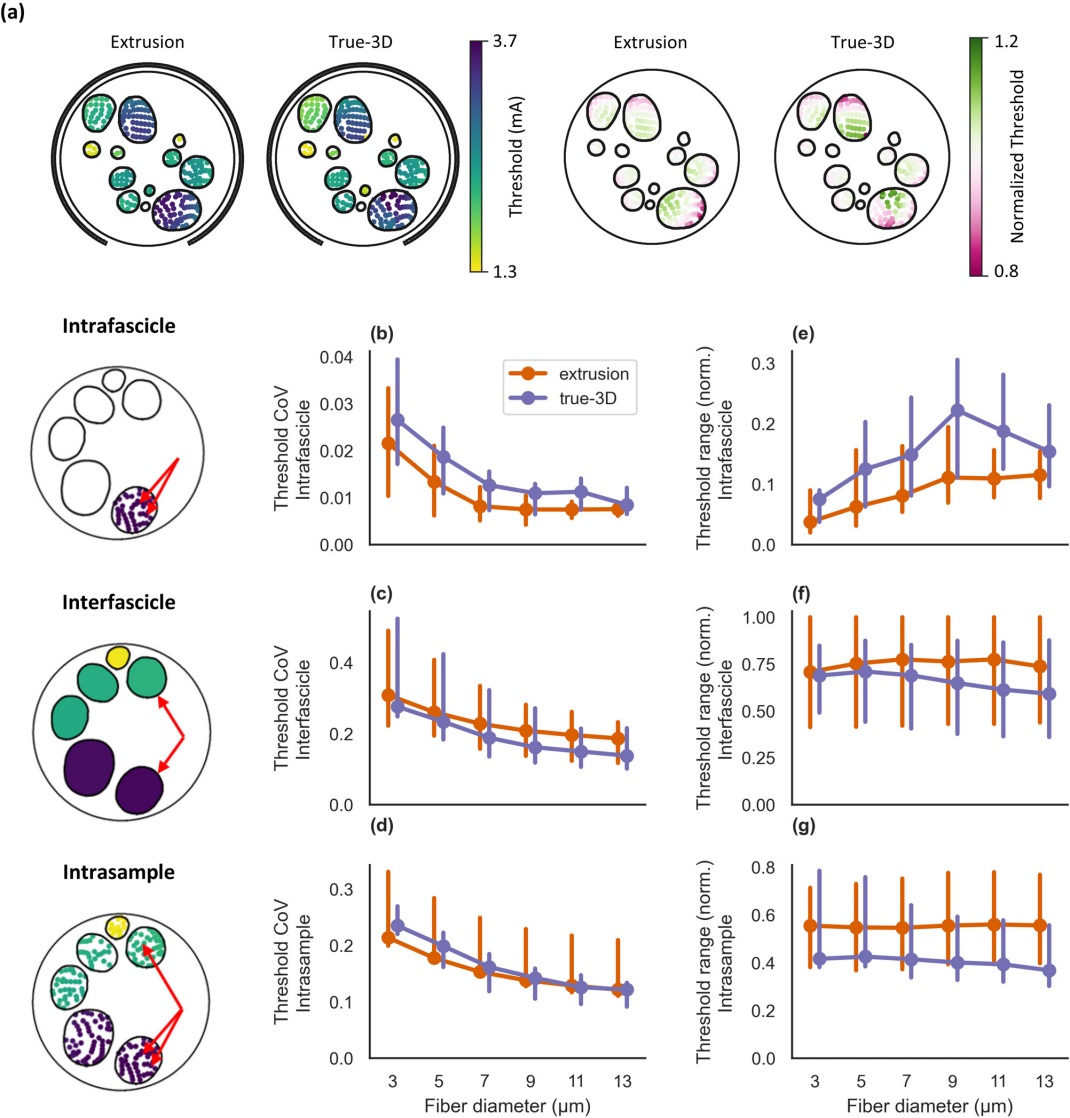
Spatial patterns of fiber activation thresholds for extrusion models (3D deformation, cathodic slice) and true-3D models. (a) Example heatmaps of activation thresholds for 3 *μ*m fibers in sample 3R: (left) raw thresholds (data for all nerves in Supplement 10) and (right) thresholds normalized by the fascicle's mean threshold. (b, c, d) Coefficient of variation for intra-fascicle (b), inter-fascicle (c), and intra-sample (d) thresholds. (e, f, g) Normalized threshold range for intra-fascicle (e), inter-fascicle (f), and intra-sample (g) thresholds. Line plots show the median with a 95% confidence interval obtained via bootstrapping. To calculate the normalized threshold range for a given analysis, we calculated the intra-fascicle, inter-fascicle, or intra-sample threshold range and divided by the maximum value across true-3D and extrusion models. Intra-fascicle data (b) and (e) were calculated as the coefficient of variation and normalized range of thresholds for each fascicle for each sample, then the median was taken across all fascicles and samples. Inter-fascicle data (c) and (f) were calculated by taking the mean threshold of each fascicle, then calculating the coefficient of variation and normalized range of the means across fascicles for each sample, then taking the median across samples. Intra-sample data (d) and (g) were calculated by taking the coefficient of variation and normalized range for all fiber locations in each sample, then taking the median across samples. We defined the fascicle morphology and fiber locations for true-3D models using the cathodic slice, as used for the extrusion models.

Extrusion model thresholds are highly homogeneous for a given fiber diameter within a fascicle^33^ [Fig. 7(a)] due to the high resistivity of the perineurium.^26^ To evaluate spatial homogeneity of activation thresholds in true-3D models, we calculated measures of threshold variation for each fiber diameter across three domains: across fibers in each fascicle [Figs. 7(b) and 7(e)], across fascicles within a nerve, with each fascicle having its fibers' thresholds aggregated [Figs. 7(c) and 7(f)], and across all fibers in each nerve [Figs. 7(d) and 7(g)]. Fiber thresholds varied more within fascicles in true-3D models [Fig. 7(b)] and more between fascicles in extrusion models [Fig. 7(c)]. Accordingly, the threshold range was larger within fascicles in true-3D models [Fig. 7(e)] and between fascicles in extrusion models [Fig. 7(f)]. Variations in fiber thresholds across the nerve were comparable between the two model types [Fig. 7(d)], but extrusion models exhibited a larger range of thresholds across all fibers in a given nerve [Fig. 7(g)]. For all comparisons, the coefficient of variation of the thresholds decreased with increasing fiber diameter [Figs. 7(b)–7(d)].

### Extrusion models can predict true-3D spatially selective activation with a multi-contact electrode

**FIG. 8.**
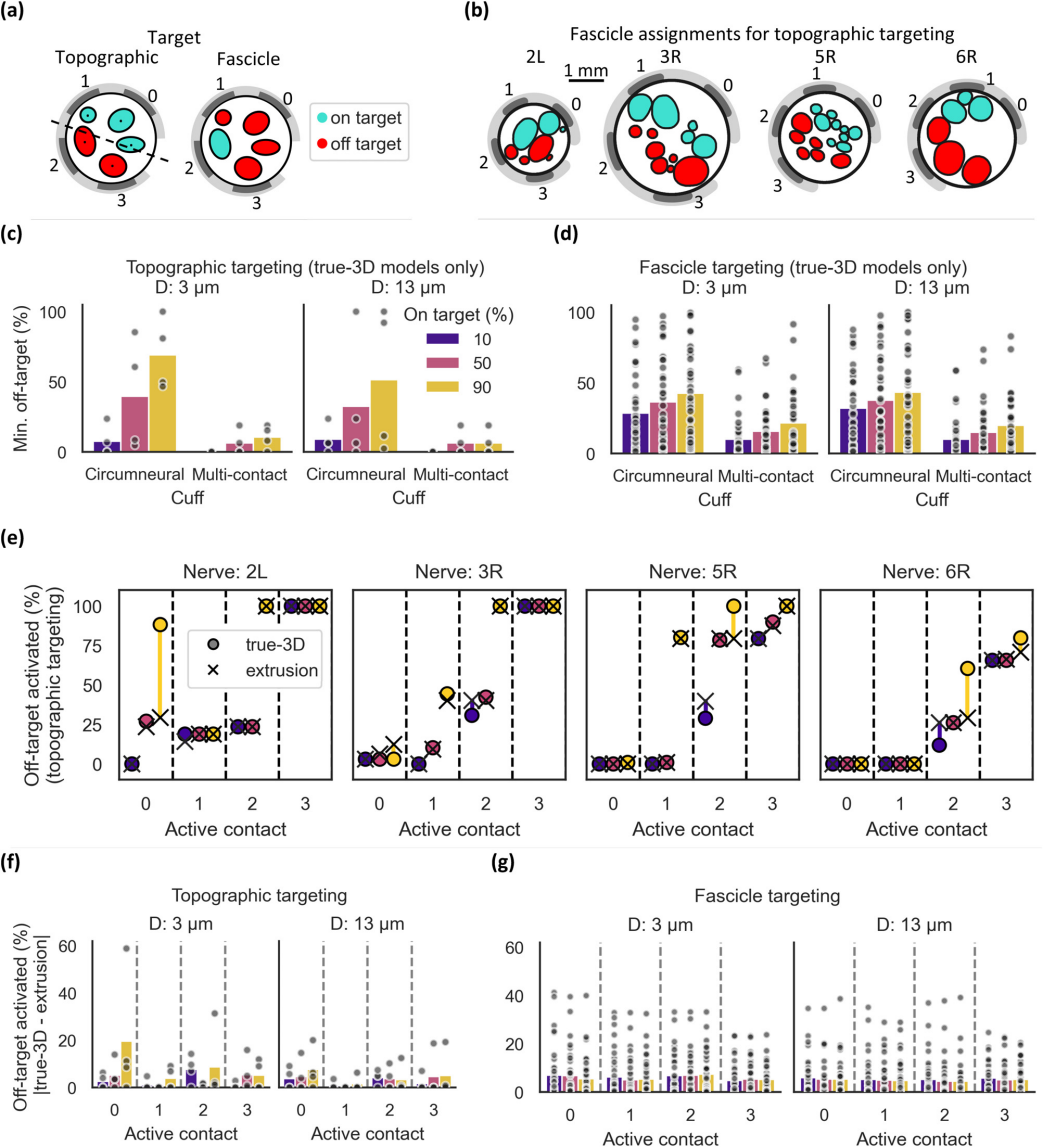
Spatially selective fiber activation using deformed true-3D and extrusion (cathodic slice, 3D deformation) models with circumneural and multi-contact cuff electrodes. (a) Diagram of the two spatial selectivity tasks, designating fascicles as on- or off-target. Under topographic targeting, all fascicles above the line bisecting the cuff geometry were designated as on-target, and all others were designated as off-target. Under fascicle targeting, each fascicle was successively designated as on-target and all others as off-target. (b) Fascicle assignments for each nerve's cathodic slice under topographic targeting. (c, d) Proportion of off-target fibers activated in true-3D models at stimulation amplitudes that activate 10%, 50%, or 90% of target fibers [topographic targeting in (c), fascicle targeting in (d)]. For the multi-contact cuff, data points denote the minimum across the four contacts that were evaluated. (e) Differences in off-target fiber activation between extrusion and true-3D models under topographic targeting. (f, g) Absolute residuals in the percent of active off-target fibers between the true-3D and extrusion models under topographic (f) or fascicle (g) targeting (bars = median).

We compared the spatial selectivity of human VNS with extrusion vs true-3D models using a custom multi-contact cuff placed at the same location on the nerve as the cathodic circumneural contact. Prior work in pigs demonstrated separation between afferent and efferent fiber groups.^14,17^ Data are emerging on the topographical organization of the human vagus nerve, but no conclusive vagotopy has been defined. Therefore, to enable comparison of selective activation between modeling approaches, we defined two spatial selectivity tasks for each nerve [Fig. 8(a)]: (1) we arbitrarily divided each nerve into “on-target” and “off-target” halves using a line perpendicular to the center of the cuff's arc length (i.e., “topographic targeting”), or (2) we assigned each fascicle as on-target, and all other fascicles off-target (i.e., “fascicle targeting”). Therefore, each nerve had one “topographic targeting” analysis [Fig. 8(b)], and a number of “fascicle targeting” analyses equal to the number of fascicles in the cathodic slice. With each of the four contacts of the multi-contact cuff electrode's center row as an active cathodic-leading monopole, we calculated the stimulation amplitude necessary to activate 10%, 50%, and 90% of on-target fibers and determined the percentage of off-target fibers that were concomitantly activated.

For each analysis, we selected the contact that activated target fibers with the lowest off-target activation. Under topographic targeting, 90% of target fibers could be activated with a median of 10.5% and 6.3% off-target fibers activated for 3 and 13 *μ*m fibers, respectively [Fig. 8(c)]. For fascicle targeting, the best contact (determined for each fascicle) activated 90% of target fibers with concomitant activation of 21.6% and 19.9% of off-target fibers (median value across all fascicles in all samples) for 3 and 13 *μ*m fibers, respectively [Fig. 8(d)].

Since true-3D models predicted highly selective activation of target fibers using a multi-contact cuff, we next compared their topographic targeting predictions to extrusion models and found that they matched well [Fig. 8(e)]. We calculated absolute percent differences in off-target activation between true-3D and extrusion models. The values were near 0 on average, indicating a strong match [Fig. 8(f)], with a median of 0.94% (range: 0%–59%) for 3 *μ*m fibers and 0% (range: 0%–20%) for 13 *μ*m fibers across all active contacts and target activation levels. For fascicle targeting, predictions of off-target activation again matched well [Fig. 8(g)], with a median difference of 2.19% (range: 0%–41%) for 3 *μ*m fibers and 2.04% (range: 0%–39%) for 13 *μ*m fibers across all active contacts and target activation levels.

Overall, off-target activation predictions for selective monopolar stimulation with deformed extrusion models using the slice under the active monopolar contact predicted well the true-3D off-target activation, although there remained outliers up to a difference of 59% between the two modeling methods.

### Symmetric bipolar stimulation requires two extrusion models per nerve

**FIG. 9.**
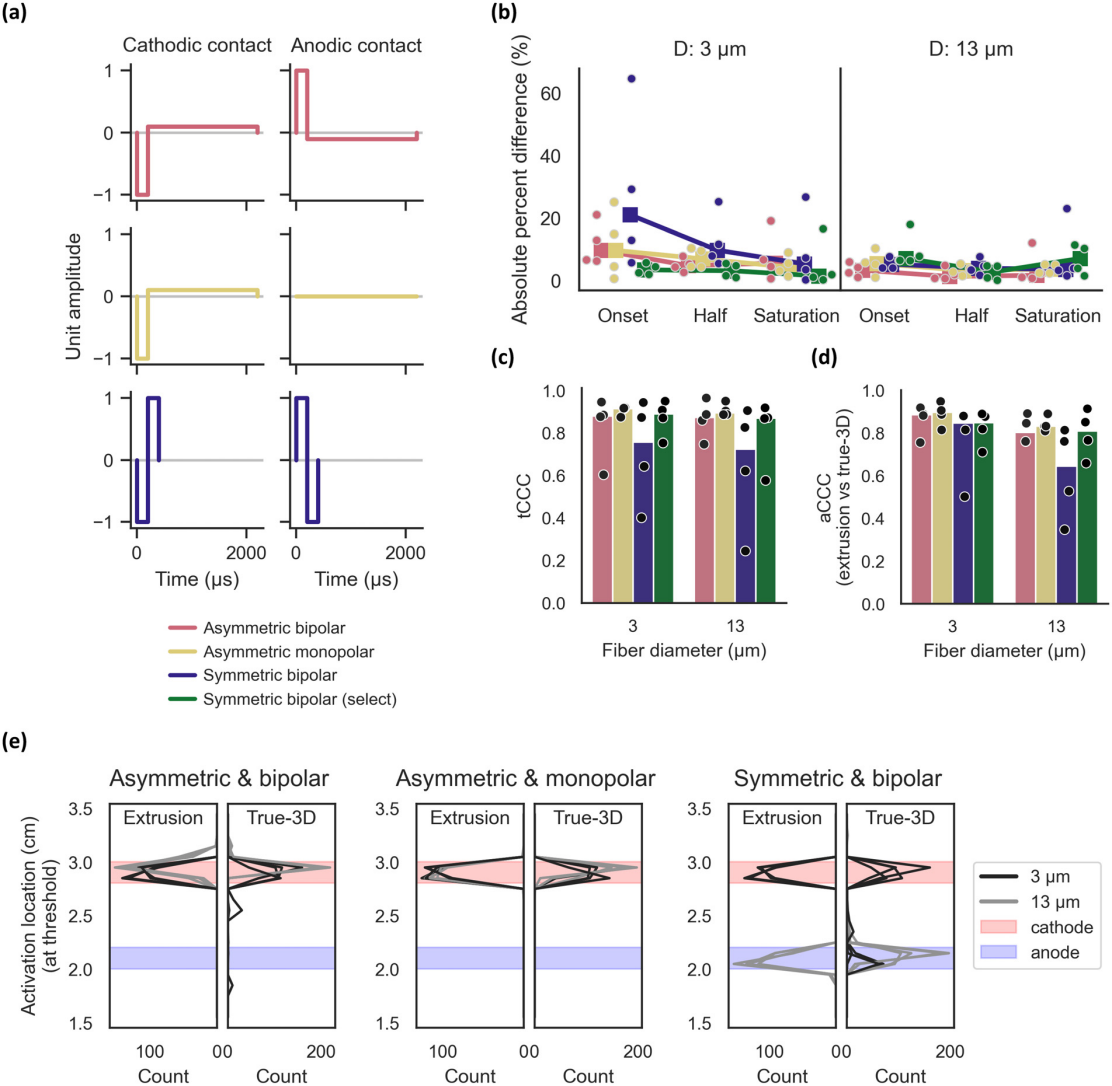
Effects of stimulation waveform on thresholds from extrusion (cathodic slice) vs true-3D models, with 3D deformation and the circumneural cuff electrode. (a) Stimulation waveforms. The waveform for “Symmetric bipolar (select)” is the same as “Symmetric bipolar.” (b) Dose-response thresholds (boxes = median). (c, d) Concordance correlation coefficient for true-3D vs extrusion model activation thresholds [tCCC, panel (c)] and activation order [aCCC, panel (d)] (bars = median). (e) Histogram of the locations of action potential initiation for each fiber in extrusion and true-3D models, defined as the z-coordinate of the node of Ranvier with the earliest rising edge of V_m_ passing −30 mV. Each nerve is a separate line.

Activation thresholds from extrusion models depend on the location of the selected nerve slice relative to the cathodic (depolarizing) contact [Fig. 3(a)]. Therefore, we assessed the effects of active contact configuration and waveform symmetry [Fig. 9(a)] on the threshold differences between extrusion and true-3D models with the circumneural cuff. Dose-response curves, fiber-specific thresholds, and fiber recruitment orders were similar between true-3D and extrusion models when using monopolar or asymmetric bipolar stimulation; however, the correspondence decreased with symmetric bipolar stimulation [Figs. 9(b)–9(d), blue vs other colors]. We determined that the locations of action potential initiation depended on the stimulation protocol [Fig. 9(e)]. In extrusion and true-3D models, activation typically occurred under the cathodic contact, with one exception: for large-diameter fibers with symmetric bipolar stimulation, anodic preconditioning reduced activation thresholds (Supplement 13), resulting in activation under the anodic contact at threshold.

With symmetric bipolar stimulation, both contacts deliver a depolarizing pulse of equal amplitude. We hypothesized that a given fiber in a true-3D model might activate locally at a different threshold near each contact, and the propagated action potentials in true-3D models would result from the contact with the lower local threshold. We simulated a hybrid approach, termed “select” symmetric bipolar stimulation. For each nerve, we implemented two extrusion models: one using the cathodic slice and one using the anodic slice. For each fiber, we then selected the lower threshold between the two extrusion models. This “select” symmetric bipolar stimulation resulted in reduced threshold differences that were comparable to asymmetric (monopolar and bipolar) stimulation [Figs. 9(b)–9(d), green vs pink and yellow).

## DISCUSSION

We implemented the first true-3D models of peripheral nerve stimulation using 3D imaging data to capture the full complexity of human vagal morphology, including fascicle merging and splitting. Previous models^19–23,34–39^ used the extrusion method due to lack of 3D imaging data and the challenges of applying the finite element method to complex biological 3D geometries. The extrusion method inherently assumes a constant nerve cross section along its length and therefore does not capture the effects of merging, splitting, and changes in transverse position of fascicles. Although a population of extrusion models of human VNS reproduced clinical dose-response data,^40^ it remained unclear whether such models accurately predict the responses of individual nerves, fascicles, or fibers. Comparing extrusion models to *in vivo* results is constrained by the fact that animal models such as pigs do not exhibit comparable complexity of vagal morphology as human nerves (i.e., fascicle splits and merges);^17^ performing such experiments in humans is impossible as it requires excising nerve tissue given current imaging methods. Furthermore, while some approaches in other nerves and species have manually reconstructed 3D nerves from histology, the complexity of the human vagus nerve makes this approach intractable for modeling human VNS.^41,42^ Therefore, true-3D models are required to evaluate the appropriateness of the extrusion assumption and thereby determine if extrusion models can adequately guide the design of human VNS therapy.

The uniquely complex fascicular morphology of the human vagus nerve^24^ poses challenges to creating true-3D models, requiring 3D structural mechanics deformation, 3D fiber pathways, and tissue anisotropy with axes different than the global coordinate system. Extrusion modeling is simpler, less error-prone, and has been automated by recently developed computational pipelines.^22,43,44^ True-3D modeling techniques can fail at several stages and require more researcher time and computational resources. Manual segmentation of a 2D cross section requires 15 minutes or less, whereas segmenting a true-3D model is a painstaking process that consumes several days. Even when 3D segmentations are automated,^45^ ensuring that the raw images are free of artifacts and that segmentations are accurate requires added researcher effort. Meshing a true-3D model in Simpleware can take days, while meshing an extrusion model typically takes under an hour. If extrusion models suffice for modeling human VNS, they can save considerable time and resources.

We implemented the first computational pipeline for modeling peripheral nerve stimulation with 3D nerve morphology, we developed a series of metrics to compare the neural responses between extrusion and true-3D models, and in n = 4 nerves, we found that extrusion models can be used for human VNS by accounting for deformation due to chronic cuff instrumentation and with appropriate slice selection. While our results were focused on cuffs placed around the vagus nerve, future studies may use our pipeline and suite of comparison metrics to quantify the effects of extruded vs true-3D nerve morphology on stimulation with inter- or intra-fascicular penetrating electrodes in the vagus or any other nerve.

### Agreement between true-3D models and extrusion models

We identified slice selection as a critical factor influencing the responses of extrusion models. Using the slice centered under the depolarizing contact was necessary for accurate dose-response thresholds [Fig. 3(b)], fiber-specific activation thresholds [Figs. 4(d) and 4(e)], and activation order [Figs. 5(c) and 5(d)]. Rapid morphological changes in the human vagus nerve cause accuracy to drop quickly when the selected slice is only a few millimeters away from the depolarizing contact: the “center” and “anodic” slices were 4 and 8 mm from the cathodic slice, respectively, and their median threshold concordance was <0.5 (vs ∼0.8 for the “cathodic” slice) [Fig. 4(e)]. Similarly, modeling pig VNS with four slices spanning 3.5 cm revealed that differences between this 3D approach and extrusion modeling increased as the extrusion slice moved further from the cuff location.^42^ A specific quantification of the relationship between loss of accuracy and the distance of the extruded slice to the cathodic contact may require a larger population of nerves given the high inter-individual variability in human vagal anatomy (Fig. 2) and stimulation responses (Supplement 14).

Given the importance of slice selection for the accuracy of extrusion models, the effects of electrode geometries and uncertainty in electrode placement must be considered. An extrusion model assumes a single known location of action potential initiation for slice selection. Thus, in the cases of multi-contact electrodes, a depolarizing contact that spans a longer length of nerve, or uncertainty in implant location or post-implant migration, modeling approaches should consider multiple extrusion models (Fig. 9) and/or true-3D models.

In addition to slice selection, modeling nerve deformation from chronic cuff instrumentation^46,47^ was also critical for extrusion model accuracy [Figs. 3(a)–3(c)]. Deformation reduced electrode-fiber distances and the amount of space between the cuff and the nerve, which is filled by saline that shunts current away from the fibers; deformation thus resulted in lower thresholds [Fig. 3(a), Supplement 8]. We hypothesize that reduced current shunting with deformation led to improved agreement between true-3D and extrusion dose-response curves by keeping current localized near the extrusion slice. 2D and 3D deformation yielded similar responses, although 3D deformed extrusion models better reproduced the onset and saturation thresholds of true-3D models [Fig. 3(c)]; this is unsurprising since 2D deformation results in a different fascicle positioning compared to 3D deformation (Supplement 15). We observed an exception wherein onset thresholds from true-3D models were better predicted by undeformed extrusion models than extrusion models with 2D deformation [Fig. 3(c)], possibly due to greater mismatch of fascicle positions between 2D deformation and true-3D models at the epineurial boundary (i.e., locations of fibers with the smallest electrode-fiber distances and thus the lowest thresholds; Supplement 15), whereas the fascicle positions are identical between undeformed extrusion models and undeformed true-3D models. 3D deformation is complex, and 3D imaging data are more challenging to obtain than histological cross sections. While 3D deformation is more realistic and resulted in extrusion models that better reproduced true-3D model thresholds, established methods of 2D deformation of extrusion models^22^ offer a practical alternative. Indeed, the 2D deformation approach in ASCENT has previously been leveraged in modeling studies that included validation against preclinical *in vivo* and clinical measurements.^14,40^

We developed the first modeled deformation of true-3D fascicular structure to mimic the conformation of the nerve to the cuff electrode that occurs with chronic implants.^46,47^ Previous studies used extrusion models, and those that accounted for deformation applied their methods to 2D cross sections by shifting the fascicles within the epineurium with no consideration of the nerve's 3D structure.^22,34^ Certain assumptions and simplifications were required for our 3D deformation given the computational challenges and limitations in available parameters. To simulate cuff-induced 3D deformation, we only used the fascicular structure, and we modeled the epineurium in each deformed region as a cylinder of constant radius, with volume matched to the undeformed epineurium. We deformed the fascicles using cylinders that were iteratively shrunk rather than the cuff electrode, and we used linearly elastic material properties for the fascicles instead of more complex, nonlinear neural tissue properties.^48^ The linear elastic properties resulted in slightly compressible fascicles, but excluding the epineurium from the simulation allowed the fascicles to displace while preserving their shapes (see “Methods: Circular deformation”) consistent with histological observations.^46,47^ For comparison, simulations of 2D deformation typically treat each fascicle as an isolated 2D physics entity.^14,22,34,38,40^ These simplifications enabled successful simulation of 3D deformation, a highly challenging modeling task: we deformed our nerves using the structural mechanics module in COMSOL v6.1, but the same approach was impossible in COMSOL v6.0. Furthermore, we excluded two nerves from this study that we could not successfully deform: in one, a large oblong fascicle under one cuff helix rendered circular deformation impossible without altering the fascicle's shape, and in another, each helix could deform individually, but attempting both simultaneously caused simulation failure for unclear reasons.

Most metrics showed better agreement between true-3D and extrusion models for larger-diameter fibers. We found that larger fibers effectively downsample the electric field and are less sensitive to local changes [Fig. 6(c)]. As a result, activation thresholds for smaller fibers are more sensitive to the morphological differences between extrusion and true-3D models. Since thresholds for large-diameter fibers already matched well between undeformed extrusion and true-3D models, deformation resulted in a larger improvement in agreement for small-diameter fibers [Fig. 4(e)], demonstrating that small fibers are more sensitive to changes in the electric field following deformation. Activation of large-diameter fibers causes the primary side effects of VNS.^9,14^ The smaller difference in thresholds between extrusion and true-3D models for larger fibers indicates that extrusion models are particularly suited to designing VNS parameters that avoid activation of these off-target fibers. Interestingly, the anode-leading pulse of symmetric biphasic stimulation preconditioned the state of the local fiber membrane and resulted in lower activation thresholds, particularly for larger fibers (Supplement 13); therefore, avoiding symmetric biphasic stimulation may reduce side effects resulting from activation of large fibers.

On average, true-3D thresholds were lower than extrusion thresholds, and the average relative percent difference was largest for small-diameter fibers [Figs. 3(d), 3(f), and 4(c)]. Several factors may reduce true-3D model thresholds: (1) tortuous fiber paths reduce thresholds;^49^ (2) fascicle splits and merges within the cuff may reduce thresholds when a fiber passes through a smaller fascicle with thinner perineurium;^24,26,27^ (3) transverse shifting of true-3D fascicles along the length of the cuff will cause the fibers within to vary their electrode-fiber distance, reducing thresholds where they pass closer to the electrode; and (4) the true-3D morphology is different under each electrode contact, and the fiber will activate at the lower threshold contact.

Extrusion models accurately predicted true-3D dose-response curves, but differences were larger for individual fascicles and fibers. Fiber-specific threshold differences were <12% on average [Fig. 4(b)], up to 150% (Supplement 9). Due to such outliers in thresholds (both high and low), thresholds to activate 50% of fibers in a given nerve resulted in the best match between true-3D and extrusion dose-response curves [Fig. 3(d)]. Considering spatial patterns of activation, thresholds within a given fascicle varied more in true-3D models (Fig. 7). Intra-fascicle thresholds vary little for extrusion models (for a given fiber diameter) because the fascicles are not interconnected and each fascicle has a constant cross-sectional area and a constant thickness of the highly resistive perineurium along its length.^23,26,33^ Because of merges and splits, fiber residency in a “fascicle” of a true-3D model is ill-defined if analysis is not restricted to a specific slice. Fascicle merges and splits in true-3D models alter local perineurium thickness and produce greater threshold variability across fibers. Furthermore, the true-3D fascicular structure in our four nerves each formed a single connected endoneurial volume, whereas fascicles in extrusion models are separate parallel entities with intervening perineurium and epineurium. Therefore, current flow through the 3D interconnected endoneurium of true-3D models generates more spatial variation in thresholds within fascicles and less spatial variation across fascicles. We did not include a direct comparison of fiber-specific responses between deformed true-3D and extrusion models with 2D deformation because their fiber locations could not be mapped one-to-one.

Across fiber locations, 3 and 13 *μ*m diameter fibers were recruited in nearly the same order for extrusion models but not for true-3D models (Fig. 6). True-3D models have greater spatial variation in the extracellular potentials of each fascicle cross section due to the complex nerve morphology, including varying fascicle locations, fascicle diameters, and perineurium thicknesses. As mentioned above, large-diameter fibers have longer distances between their nodes of Ranvier and are less affected by small spatial variations through downsampling of the electric field. Smaller fibers, with less downsampling, are more sensitive to local variations in the field that may increase excitability [Fig. 6(c)]. The change in internodal distance and therefore different downsampling of the electric field causes changes in the excitability of fibers following the same path. To ensure there were not systematic differences in excitability due to node of Ranvier alignment relative to the cuff electrode, we randomly shifted the longitudinal alignment of each fiber. Although extrusion models predict that fibers of the same diameter will activate in a sequence largely determined by electrode-fiber distance and perineurium thickness, true-3D models exhibited changes in recruitment order depending on the selected fiber diameter.

### Spatial selectivity and multi-contact stimulation

Spatially selective stimulation can reduce side effects by avoiding activation of off-target fibers and increase therapeutic effects through greater activation of target fibers.^7,8,11,12^ Given the ongoing development of multi-contact cuff electrodes to enable selective stimulation,^14,19,50^ we supplemented our simulations of the standard clinical cuff—which has circumneural metal contacts—with simulations using a multi-contact cuff. Our multi-contact cuff electrode enabled robust selective activation of fibers with monopolar stimulation, comparable on average for true-3D vs extrusion models, despite the aforementioned differences in spatial patterns of activation. Extrusion models reproduced well the selectivity predicted by true-3D models, both when we designated half the nerve as on-target and half as off-target, as well as when we designated one fascicle as on-target and the rest of the nerve as off-target. These results provide a foundation to leverage emerging data on human vagal anatomy, including spatial organization of fibers with different functions and diameters.^15,17,18^

Using a multi-contact cuff electrode with small metal contacts placed around the circumference of the nerve generally produced a closer match between extrusion and true-3D fiber-specific thresholds and activation order than the electrode with circumneural contacts [Figs. 4(f) and 5(e)]. Therefore, extrusion model predictions of selective stimulation are likely to be more accurate when using small electrode contacts that can spatially target neural activation. We hypothesize that this improved match results from an increased dominance of electrode-fiber distance on activation thresholds when using the multi-contact cuff. For the circumneural cuff, most fibers have small electrode-fiber distances, with only those few fibers in the center having a large electrode-fiber distance. For the multi-contact cuff, the range of electrode-fiber distances is doubled (the diameter, rather than the radius of the nerve), and they will be spread more evenly throughout the range. This may lead to the observed increase in concordance of specific fiber thresholds throughout each model nerve when using the multi-contact cuff.

Compared to the circumneural cuff models, multi-contact cuff extrusion models and true-3D models had less agreement of half and saturation dose-response thresholds and stronger agreement of onset thresholds [Fig. 3(e)]. In the true-3D models, fascicles can merge, split, and shift positions, allowing current to flow among them in ways not captured by the extrusion models, where each fascicle is isolated and has a constant transverse position. We hypothesize that as distance from the active multi-contact electrode increases, morphological differences in the true-3D models accumulate and produce greater variability in the electric field; this could explain why saturation thresholds—reflecting activation of the most distant fibers—show the largest discrepancy between extrusion and true-3D multi-contact cuff models.

Our multi-contact simulations used only the middle row of contacts, simulating a scenario where the depolarizing contacts are aligned with the cross-sectional slice used in the extrusion models. This setup favors a strong match between the predictions of extrusion and true-3D models. In many models of peripheral nerve stimulation, cuffs have multiple contacts arranged along different longitudinal positions to enable current steering and improve spatial selectivity.^14,42,51^ In these cases where cuffs span a large longitudinal extent of the nerve—with multiple depolarizing contacts at different longitudinal locations—a single extrusion model is unlikely to generate accurate nerve responses; our results suggest that a different extrusion model for each longitudinal position of electrode contacts could accurately replicate the fiber recruitment predicted by a full true-3D model (Fig. 9).

### Implications of imaging methods for anatomical accuracy and segmentation in true-3D models

3D imaging to parameterize true-3D models of peripheral nerve stimulation is challenging because it demands both high isotropic spatial resolution and sufficient contrast to resolve the primary neural tissues. In this study, we leveraged the first-ever 3D imaging of human vagus nerve morphology^24^ to build highly realistic true-3D models of VNS.

Perineurium thickness has a large effect on modeled thresholds.^26^ Segmenting the perineurium was not possible because osmium tetroxide staining does not produce distinct perineurial contrast. Therefore, we relied on the linear relationship between perineurium thickness and fascicle diameter,^27^ as used in previous modeling efforts.^14,40^ In our true-3D models, we generated perineurium around fascicles on a slice-by-slice basis, rather than as a 3D tissue. We expect that our approach approximates true vagal morphology since histology suggested that upon a branch event, the perineurium rapidly reorganizes to a new thickness (Supplement 17). We also could not resolve individual fibers using our imaging data. Therefore, we developed a method to place fibers throughout the endoneurium based on its contours. Along these pathways, we simulated myelinated fibers because the stimulation amplitudes required to activate unmyelinated fibers are much greater than what is delivered in clinical VNS.^9,52^ Future work may pair specific fiber positioning derived from immunohistochemistry with 3D imaging of the nerve to place nerve fibers within true-3D morphology, increasing model realism.

It was challenging to resolve and segment the smallest fascicles in our microCT imaging—particularly as some fascicles of the human vagus nerve only contain a few fibers^53^—and our streamline-based method of defining 3D fiber pathways sometimes failed to place fibers in the smallest segmented fascicles. Given the relative size of these fascicles, they would have a small contribution to the nerve response by fiber count, and their exclusion is unlikely to change our conclusions; however, their inclusion could lead to a slightly lower onset response because fibers in smaller fascicles have lower activation thresholds.^26^

## CONCLUSIONS

VNS is an established therapy for conditions refractory to conventional treatments, yet maximizing efficacy while minimizing side effects remains a challenge. Computational modeling enables the design of VNS parameters and contributes to understanding the underlying mechanisms of action. We addressed the substantial longitudinal morphological variability of the human vagus nerve by implementing the first true-3D simulation of peripheral nerve stimulation using segmentations from volumetric imaging. We developed a comprehensive suite of metrics—including population-level responses, fiber-specific thresholds, spatial patterns of activation, and selectivity—to compare systematically true-3D model predictions with those from conventional extrusion models. Extrusion models—which are much less complex to construct—closely matched the neural activation predicted by true-3D models given two conditions: (1) nerve deformation from chronic cuff instrumentation and (2) selection of an extrusion slice centered underneath the depolarizing contact. Even shifting the extrusion slice a few millimeters from this contact substantially increased the discrepancy in activation thresholds. Consequently, extrusion modeling requires that the cross-sectional placement of the contact is precisely known. When these conditions are not met, true-3D modeling is needed to capture the full complexity of fascicular merging, splitting, and reorganization. The true-3D modeling workflow presented herein is a substantial technical advance for realistic modeling of peripheral nerve stimulation and lays a foundation for patient-specific modeling and optimization of vagus nerve stimulation.

## METHODS

Figure 1(a) shows our workflow for modeling VNS with true-3D nerve morphologies. We implemented anatomically realistic models of four human vagus nerves, each derived from a different cadaver. We created true-3D models of nerves using segmented microCT images, and we selected slices (i.e., cross sections) from the true-3D models to create extrusion models of the same nerves [Fig. 1(b)]. ASCENT is an open-source pipeline for extrusion modeling of peripheral nerve stimulation;^22^ we adapted ASCENT to implement true-3D finite element models, used ASCENT v1.2.2^54^ to implement extrusion finite element models, and used PyFibers v0.1.1 to simulate the responses of biophysical nerve fibers.^55^

### Nerve sample acquisition, imaging, and segmentation

As part of a previous study,^24^ we collected human cervical vagus nerves from de-identified cadavers donated to the Case School of Medicine Anatomy Department. This work was determined to be exempt from human subjects research by Case Western Reserve University's Institutional Review Board (IRB) because it involved de-identified cadaveric tissue and no protected health information was collected. The imaging methods are described in detail in our previous work,^24^ and briefly herein.

#### MicroCT imaging

We dissected and removed the nerves from the nodose ganglion to the clavicle (11.5–18 cm in length; mean length of 14 cm). Each nerve was labeled with the subject number followed by the letter R (right) or L (left) to indicate the side of the body (total n = 4 samples labeled 2L, 3R, 5R, and 6R). We stained the samples with 1% (vol./vol.) osmium tetroxide solution (Polysciences, IL, USA) as previously described.^24^ We placed each nerve in a plastic mold and filled the mold with paraffin; the plastic mold had grooves every 5 mm marked with acrylic paint enriched with barium sulfate (Sigma Aldrich) to facilitate navigation during imaging. We imaged each nerve using a Quantum GX2 microCT scanner (PerkinElmer, Waltham, MA, USA) with a scan voltage of 90 kV, a current of 80 *μ*A, and a 14 min scan duration. The scan filter consisted of Cu 0.06 mm + Al 0.5 mm (PerkinElmer). The cross-sectional field of view was 36 mm. We imaged the middle 5 cm of each nerve in 1.8 cm-long longitudinal segments with ∼0.3 cm (17%) overlap between neighboring segments.

We performed image reconstruction using the Rigaku software (PerkinElmer). Each ∼0.5 cm-long segment of the reconstructed image had 10 *μ*m isotropic voxels, exported as a stack of 16-bit TIFF images (512 × 512 × 512 pixels). To simplify image post-processing and segmentation, we downsampled the dataset along the z-dimension by a factor of 10, yielding 51 TIFF images (512 × 512 × 51 pixels). We stitched the overlapping volumes using ImageJ (FIJI, v2.1.0/1.53c). Voxel dimensions were thus 10 × 10 × 100 *μ*m^3^ in the images used for segmentation, with the lower resolution along the longitudinal z-axis. The final length of each nerve sample was 5.01 cm (501 xy image slices). For one microCT image (sample 5R), there was transverse shifting between slices due to sample movement between acquisition scans, which we resolved using non-rigid motion correction (Supplement 1).

#### Segmentation

We imported the reconstructed, downsampled microCT images into Simpleware™ ScanIP (version T-2022.03 with FE and CAD add-ons, Synopsis, Sunnyvale, CA, USA). We manually segmented the fascicles (endoneurium) using built-in perimeter tracing tools in Simpleware, excluding fascicles that crossed outside of the epineurium. We manually segmented the epineurium every 10th slice and used Simpleware's interpolation tool to segment the epineurium in the remaining slices. For the fascicles, we segmented every slice. Because there was drift in the transverse position of the nerve throughout each sample, we centered each transverse slice on the centroid of the segmented epineurium (i.e., the mean location of white pixels in the binary image). After centering, we used Simpleware's de-stepping smoothing on the segmented epineurium and fascicles. We converted the resulting smoothed surfaces into masks and upsampled 5× in the z-direction using linear interpolation. The de-stepping operation could cause erroneous merging of neighboring fascicles, which we manually separated using the Simpleware paint tool. Some fascicles that moved quickly in the xy-plane between slices were erroneously reduced in volume by the de-stepping; we re-segmented those fascicles using the Simpleware paint and interpolation tools while visualizing the segmentations overlaid on the raw microCT image. Finally, to ensure compatibility with finite element modeling, we applied multiple preprocessing steps to the segmentations (Supplement 2) before binarizing the fascicle and epineurium masks and exporting each as a stack of TIFF images.

#### Shrinkage correction and perineurium

We applied 2D isotropic rescaling to each slice with a scale factor of 1.2 (20% inflation) to correct for tissue shrinkage during sample processing.^56,57^ We defined the perineurium thickness for each fascicle in each slice based on the fascicle diameter, as reported previously:^27^

$$thk_{\text{peri}}=0.037\;02*d_{\text{fasc}}+10.50,$$ (1)

where thk_peri_ is the thickness of the perineurium in *μ*m and d_fasc_ is the effective circular diameter of the fascicle in *μ*m (i.e., the diameter of the circle with the same cross-sectional area as the original fascicle mask), given by

$$d_{\text{fasc}}=2*\sqrt{\frac{A_{\text{fasc}}}{\pi }},$$ (2)

where A_fasc_ is the area of the given fascicle. For the true-3D models, rapid movement of fascicles transversely across slices could result in small areas of “exposed” endoneurium. We developed an algorithm—detailed in Supplement 3—that ensured that all endoneurium was enclosed with perineurium of the appropriate thickness according to Eq. (1).

### Finite element models of electric currents

#### Cuff geometry

We modeled the bipolar circumneural cuff electrode (LivaNova PLC, London, United Kingdom) that is used clinically to treat epilepsy and depression as previously implemented^40^ [Fig. 1(c)]. Given the potential of multi-contact cuffs to stimulate selectively fibers in the vagus nerve, we also implemented a custom 12-contact cuff electrode. The custom multi-contact electrode [Fig. 1(c)] had a 5.4 mm long, 1 mm thick cylinder of silicone as insulation and three rows of four circular platinum contacts (1 mm diameter): one row at the longitudinal center of the insulation and the other two rows at ±1.35 mm (center-to-center). The four contacts in each row were placed at 45°, 135°, 225°, and 315°, where the slit in the insulation opens at 0°. We centered each cuff on the nerve centroid and then set the rotation of the cuff: for the circumneural cuff, the center of the cuff's rotational arc (center of the metal contact) was at 90° (x = 0, +y), and for the multi-contact cuff, the insulation extended from 0° (y = 0, +x) around the nerve by its expanded arc length. The rotation of each nerve was arbitrary but reproducible, such that the rotation was always the same for a given nerve.

We placed the clinical cuff halfway along the length of each nerve, conforming each helix to the minimum bounding cylinder of the true-3D nerve epineurium within its length [Figs. 1(a) and 3(a); see “Methods: Circular deformation”]. We aligned the multi-contact cuff at the center location of the caudal clinical cuff contact, conforming it to the same cylindrical boundary. Note that the multi-contact cuff consisted of only one geometry, rather than two discrete geometries as with the clinical cuff's two helices. We parameterized both cuffs to expand to accommodate a nerve larger than their resting inner diameters of 2 or 3 mm, with a minimum separation of 100 *μ*m between the inner surface of the cuff and the epineurium. The clinical cuff expanded by unfurling, and the multi-contact cuff expanded at its slit in the insulation. For one nerve (sample 2L), the minimum bounding cylinder for both helices had a diameter of <3 mm, so we used the model with a resting inner diameter of 2 mm; for all other nerves, we used the cuffs with a resting inner diameter of 3 mm. We used the same cuff model for each nerve across the undeformed and deformed models. We enclosed each helix of the clinical cuff and the entire multi-contact cuff with a volume of saline extending 100 *μ*m in every direction.

To conform each helix of the cuff to the true-3D models, we first calculated the union of all epineurium boundaries under the cuff span. We then used ASCENT to calculate the x–y location and radius of the minimum bounding cylinder; the cuff was centered at this x–y location, and its internal radius was set to the minimum bounding radius plus a gap of 100 *μ*m. For each extrusion model with either the clinical cuff or the multi-contact cuff, we matched the cuff's transverse location and conformation (i.e., epineurium bounding cylinder) from the corresponding true-3D model, and we placed the cuff at the longitudinal center of the extrusion model. For extrusion models generated using the slice centered between the two clinical contacts, we calculated the minimum bounding cylinder from true-3D models as if there were a third helix placed at the longitudinal center of the sample.

#### Circular deformation

Nerves deform to accommodate the shape of the cuff electrode, where fascicles are repositioned within the cross section of the nerve.^46,47^ We deformed the true-3D models to a circular cross section at the location of each clinical cuff helix. Prior studies using extrusion models relied on shifting fascicles in a single cross section to model deformation^22,34^ and were unable to consider 3D structural mechanics of the interconnected fascicles, as nerves had not undergone 3D imaging. To model 3D deformation, we used a 3D structural mechanics approach with linear elastic materials; this approach accounted for 3D fascicle connectivity and repositioned fascicles without changing their shape or size, as observed in histological studies.^46,47^

Our 3D deformation methods are described in detail in Supplement 4. Briefly, we truncated the perineurium geometry (perineurium and endoneurium as one merged domain) to ±2.5 mm beyond the longitudinal span of the two clinical cuff helices. We placed a cylindrical “compressor” geometry equal in length (5.4 mm) and longitudinal position to each of the two helices of the clinical cuff electrode. We fixed the ends of the perineurium in space. We assigned linearly elastic material properties to the compressors (E = 1 × 10^11^ Pa, ρ = 3900 kg/m^3^, ν = 0.222) and perineurium (E = 1 × 10^5^ Pa, ρ = 3000 kg/m^3^, ν = 0.3). We comment on the appropriateness of these material properties in the Discussion. For each compressor, we calculated the mean equivalent cylindrical diameter (ECyD) of the nerve geometry under its longitudinal extent:

$$\text{ECyD}=\frac{2}{n}*\sum_{i=1}^{n}\sqrt{\frac{A_{i}}{\pi }},$$ (3)

where A_i_ is the area of the nerve slice at index i. The slices were spaced by 20 *μ*m, and each compressor spanned 270 slices (5.4 mm). We conducted a structural mechanics simulation in COMSOL Multiphysics v6.1 (COMSOL, Burlington, MA) to shrink the compressors iteratively to ECyD minus 50 *μ*m (starting from 2× this final diameter); the 50 *μ*m buffer ensured that the fascicles would be fully enclosed by the new cylindrical epineurium.

We redefined the epineurium as a cylinder with a circular cross section using ECyD within the span of the compressors and then transitioned the epineurium from purely circular to fully undeformed over a 2.5 mm distance on either side of the clinical cuff span. We regenerated the endoneurium by inverting Eq. (1).

To implement extrusion models with deformation, we either (1) used slices from the deformed true-3D model or (2) used ASCENT's 2D physics-based deformation algorithm on slices from the undeformed true-3D model. For the multi-contact models, we placed the multi-contact cuff centered longitudinally within the section that was deformed for the caudal clinical cuff helix.

#### Assembled geometry and meshing

We used different meshing strategies for the extrusion and true-3D models because the complex geometries of the true-3D models failed to mesh in COMSOL, but we have an established pipeline for extrusion models using COMSOL.^22^ Therefore, we used COMSOL to mesh the extrusion models and Simpleware to mesh the true-3D models. We used our previous convergence analysis to determine mesh parameters and model dimensions:^40^ as specified below, we bounded the mesh element size from 10 to 1600 *μ*m, used a medium, and assigned the material properties of muscle (Table I) 10 mm in diameter and 51 mm in length. We verified that the Simpleware meshing generated the same results as COMSOL meshing for an extrusion model (Supplement 5) and that activation thresholds did not change when we refined the mesh resolution in Simpleware (Supplement 6). During all threshold searches (see “Methods: Cable models of nerve fibers”) we verified that no end excitation occurred.

**TABLE I.** Conductivity values for the tissues and materials in the finite element models. Conductivities for the endoneurium and muscle are isotropic in the transverse (xy) plane and higher along the nerve (z axis).

Material	Conductivity (S/m)	References
Silicone	1 × 10^–12^	58
Platinum	9.43 × 10^6^	59
Endoneurium	(1/6, 1/6, 1/1.75)	33, 60
Epineurium	1/6.3	23, 61, 62
Muscle	(0.086, 0.086, 0.35)	63
Saline	1.76	64
Perineurium	1/1149	33, 65

For the true-3D models, we imported our processed epineurium, perineurium, and endoneurium masks into Simpleware and applied the mesh preprocessing steps described in Supplement 2. We used COMSOL to create geometries for the cuff geometry, saline surrounding each cuff helix, and surrounding medium and exported them as STLs, which we also imported into Simpleware. We converted the saline domain around each cuff electrode to a mask to simplify meshing but left the cuff geometry and medium as surfaces. We remeshed all surfaces, and we added all masks and surfaces to a finite element model, meshed with quadratic free tetrahedral elements (“minimum edge length”: 0.01 mm, “max error”: 0.005 mm, “max edge length”: 1.6 mm, “internal change rate”: 30, “surface change rate”: 60, “n layer elements”: 1.0, “smooth against background”: true, “second order”: true). We exported the meshed model as a NASTRAN file for import into COMSOL. Mesh element counts for true-3D models ranged from ∼39 to 109 × 10^6^, with a mean of 67 × 10^6^. Mesh element counts for extrusion models ranged from ∼10 to 40 × 10^6^, with a mean of 25 × 10^6^.

For the extrusion models, we used ASCENT to implement each finite element model in COMSOL. For each true-3D nerve, we implemented three extrusion models for each deformation condition: the slices under the cathodic and anodic clinical contacts, as well as the slice centered longitudinally between the contacts. We selected these slice locations for two reasons. First, we wanted to capture the likely locations of action potential initiation, as well as a midpoint. Second, we wanted to characterize differences in true-3D and extrusion model responses along the length of the clinical cuff span. We extruded each nerve slice 50 mm longitudinally (z-direction) and placed either the clinical or multi-contact cuff on the nerve. We placed the nerve and cuff in a cylindrical domain (10 mm in diameter, 51 mm in length, shifted in the z-direction by 0.5 mm to enclose the nerve). We meshed the extrusion models with quadratic free tetrahedral elements (“curvature factor”: 0.2, “minimum element size”: 10 *μ*m, “maximum element size”: 1600 *μ*m, “resolution of narrow regions”: 1, “maximum element growth” rate: 2.2).

#### Electrical parameters and solving

We assigned boundary conditions, tissue properties, and material properties in COMSOL [Table I and Fig. 1(b)]. The conductivity of the endoneurium is anisotropic.^33^ Therefore, in the extrusion models, we applied the vector defining the conductivity of the endoneurium using the global xyz Cartesian coordinate system, with the nerve slice in the xy-plane and higher endoneurium conductivity along the z-axis. For true-3D models, we conducted a curvilinear coordinates diffusion simulation in COMSOL to define flow vectors through the endoneurium, defining the endoneurial surfaces on the rostral and caudal ends of the nerve as inlets and outlets, respectively. The curvilinear coordinates served two purposes: (1) to define the local coordinate system for assigning anisotropic conductivity to the endoneurium, and (2) to define the 3D fiber pathways along which the electrical potentials are sampled.

We placed a point current source at the center of each metal contact, and we grounded the outer boundary of the model.^66^ We solved Laplace's equation using quadratic solution shape functions for each electrode contact in each model; for each solution we set one point current source to 1 mA and all others to 0 mA (inactive). Current was allowed to flow through each physical contact, even if the point current source within was inactive.

To define the coordinates of the fiber paths in the true-3D models, we generated streamlines from the curvilinear coordinates solution described above (positioning: “on selected boundaries,” number: 250, selection: caudal endoneurium boundaries, smoothing: “inside material domains,” integration tolerance: 0.0001). We excluded streamlines that did not extend the full length of the nerve.

We sampled the potentials from the solved finite element models every 1 *μ*m along each fiber pathway. To define the coordinates of the fiber paths in extrusion models, we used the xy-coordinates from the 3D streamlines at the longitudinal position of the nerve slice used for that extrusion model. This enabled direct comparison of fiber-specific responses between the deformed true-3D models and 3D deformed extrusion models, as well as between the undeformed true-3D and extrusion models. However, the meshing approaches in the extrusion vs true-3D models resulted in slight differences between their cross-sectional morphologies; therefore, if a fiber from the true-3D model was outside of the perineurium in the associated extrusion model, we moved it to 5 *μ*m inside the fascicle's inner perineurium boundary. When we applied ASCENT's 2D deformation algorithm for extrusion models, we tracked each fiber location during deformation and, where relevant, applied this same correction.

### Cable models of nerve fibers

We used ASCENT^22^ and PyFibers^55^ with NEURON v8.2.2^55,67^ and Python 3.11^68,69^ to simulate MRG-interpolation (McIntyre–Richardson–Grill) mammalian myelinated fiber models^22,70^ with diameters from 3 to 13 *μ*m. Each fiber spanned the full nerve length of the nerve samples, and we assigned a random longitudinal alignment to each fiber between 0 and half of the internodal length. All 3 *μ*m fibers had 178 or 179 nodes of Ranvier in extrusion models and 179–190 nodes in true-3D models; all 13 *μ*m fibers had 36 or 37 nodes of Ranvier in extrusion models and 36–38 nodes in true-3D models. We linearly interpolated the sampled potentials at the center of each fiber compartment. For both extrusion and true-3D models, we calculated the electrical potentials as linear sums of the finite element solutions multiplied by a weight of −1, 0, or 1 to represent the polarity of each contact.

To help avoid end excitation, we modeled the node of Ranvier at the ends of each fiber as passive (g_m_ = 0.0001 S/cm^2^, c_m_ = 2 *μ*F/cm^2^, V_rest_ = −70 mV). We simulated each fiber at rest from t = −200 ms to t = 0 ms with a time step of 10 ms to ensure steady state at t = 0. At t = 1 ms, we applied the potentials to the fiber using a charge-balanced biphasic asymmetric waveform with a primary phase of 200 *μ*s; we approximated the passive recharge of the LivaNova implantable pulse generator with a 2 ms rectangular pulse of opposite sign and equal area [Fig. 1(d)]. Each simulation ended at t = 50 ms and was solved with backward Euler integration using a time step of 1 *μ*s.

We quantified activation thresholds using a bisection search. We defined action potential detection as a rising edge of the transmembrane potential at −30 mV at the node of Ranvier closest to 90% of the fiber's length (i.e., the caudal end). We terminated the bisection search when the top (activation) and bottom (no activation) current amplitudes were within 1% of each other. We instructed PyFibers to raise an exception in the event of end excitation, which did not occur for any threshold searches.

### Analysis

We used Python 3.11^69^ to quantify the differences in neural recruitment between true-3D and extrusion models across several metrics. We compared activation thresholds for three different deformation conditions: (1) undeformed true-3D vs undeformed extrusion models, (2) true-3D models deformed using structural mechanics (“deformed true-3D model”) vs extrusion models deformed using 2D physics-based deformation (“2D deformation”), and (3) deformed true-3D models vs extrusion models created using slices from the corresponding deformed true-3D models (“3D deformation”). For each true-3D model, we implemented three extrusion models using different slices: at the center of each of the two helical electrode contacts [termed “cathodic” and “anodic” slices based on the primary phase of the stimulation pulse: Fig. 3(a)] and halfway between the two contacts (termed “center”).

We calculated dose-response curves as the proportion of activated fibers as a function of the stimulation amplitude for each model and fiber diameter. We defined three levels of activation to characterize the population response: we denoted the stimulation amplitude required to activate 10%, 50%, and 90% of fibers, respectively, as onset, half, and saturation.

We calculated the relative and absolute percent differences between each pair of thresholds from extrusion and true-3D simulations

$$\text{Relative percent difference }=\Delta_{\text{rel}}\%=\frac{T_{\text{extrusion}}-T_{\text{true}-3D}}{T_{\text{true}-3D}}\times 100\%,$$ (4)

$$\text{Absolute percent difference }=\Delta_{\text{abs}}\%=\left|\frac{T_{\text{extrusion}}-T_{\text{true}-3D}}{T_{\text{true}-3D}}\right|\times 100\%.$$ (5)

We used Lin's concordance correlation coefficient (CCC)^31^ to assess the match between thresholds in the two models (threshold CCC; tCCC). This is distinct from Pearson's correlation coefficient, which simply measures covariance of the data, while the CCC measures how much the data values of one metric match the data values of another. Because the CCC penalizes deviations from the identity line, it provided a measure of how well extrusion-derived thresholds agreed with those from true-3D simulations. Values close to 1 signified that the fiber thresholds from the two models were similar, whereas lower values indicated less agreement. We also used the CCC to analyze fiber activation order (activation CCC; aCCC). For each fiber in an extrusion or true-3D model, we determined the fraction of fibers activated below its threshold, thus defining its activation rank. We computed the aCCC to compare the recruitment ranks of each fiber in extrusion vs true-3D models. Values close to 1 signified that the two models agreed on the order in which fibers were recruited, whereas lower values indicated different patterns of recruitment order.

Throughout these analyses, we used bootstrapping to estimate 95% confidence intervals using the default arguments for error bars in seaborn [errorbar = (“ci,” 95), n_boot = 1000, seed = None].

## SUPPLEMENTARY MATERIAL

See the supplementary material for the following: (1) motion correction, (2) segmentation preprocessing, (3) end-caps for exposed endoneurium, (4) deformation of true-3D nerves, (5) meshing extrusion models in Simpleware versus COMSOL, (6) mesh convergence in Simpleware, (7) morphological metrics for all nerve samples, (8) dose-response curves for each sample, (9) absolute and relative percent differences for all fibers, (10) threshold and activation order heatmaps for all nerves, (11) activation order plots for each nerve, (12) across-diameter comparisons of activation order, (13) thresholds across stimulation paradigms, (14) dose-response curves across individuals, (15) comparison of deformation results, (16) second differences for all 3 and 13 *μ*m fibers in each sample, and (17) perineurium around merges and splits.

## Data Availability

The computational pipeline code that underlies the findings of this study is openly available from GitHub at https://github.com/wmglab-duke/3D_nerve_pipeline; and Zenodo at Ref. 71. The data and plotting code that support the findings of this study are openly available from SPARC.science at Ref. 72.
